# Unsupervised learning and natural language processing highlight research trends in a superbug

**DOI:** 10.3389/frai.2024.1336071

**Published:** 2024-03-21

**Authors:** Carlos-Francisco Méndez-Cruz, Joel Rodríguez-Herrera, Alfredo Varela-Vega, Valeria Mateo-Estrada, Santiago Castillo-Ramírez

**Affiliations:** ^1^Programa de Genómica Computacional, Centro de Ciencias Genómicas, Universidad Nacional Autónoma de México, Cuernavaca, Mexico; ^2^Programa de Genómica Evolutiva, Centro de Ciencias Genómicas, Universidad Nacional Autónoma de México, Cuernavaca, Mexico

**Keywords:** natural language processing, topic modelling, *Acinetobacter baumannii*, antibiotic resistance, nosocomial pathogens, ESKAPE pathogens, text mining

## Abstract

**Introduction:**

Antibiotic-resistant *Acinetobacter baumannii* is a very important nosocomial pathogen worldwide. Thousands of studies have been conducted about this pathogen. However, there has not been any attempt to use all this information to highlight the research trends concerning this pathogen.

**Methods:**

Here we use unsupervised learning and natural language processing (NLP), two areas of Artificial Intelligence, to analyse the most extensive database of articles created (5,500+ articles, from 851 different journals, published over 3 decades).

**Results:**

K-means clustering found 113 theme clusters and these were defined with representative terms automatically obtained with topic modelling, summarising different research areas. The biggest clusters, all with over 100 articles, are biased toward multidrug resistance, carbapenem resistance, clinical treatment, and nosocomial infections. However, we also found that some research areas, such as ecology and non-human infections, have received very little attention. This approach allowed us to study research themes over time unveiling those of recent interest, such as the use of Cefiderocol (a recently approved antibiotic) against *A. baumannii*.

**Discussion:**

In a broader context, our results show that unsupervised learning, NLP and topic modelling can be used to describe and analyse the research themes for important infectious diseases. This strategy should be very useful to analyse other ESKAPE pathogens or any other pathogens relevant to Public Health.

## 1 Introduction

Antimicrobial drug resistance is a major public health problem. In this regard, *A. baumannii* —member of the ESKAPE group: *Enterococcus faecium*, *Staphylococcus aureus*, *Klebsiella pneumoniae*, *Acinetobacter baumannii*, *Pseudomonas aeruginosa*, and *Enterobacter species* – is a nosocomial pathogen among the most important bacterial pathogens (Ikuta et al., 2022). The World Health Organization has listed this pathogen as the highest-priority bacterium for which novel antibiotics are required (World Health Organization, 2017). Given its clinical relevance, much of the research efforts about this bacterium have focused on clinical isolates. However, no proper study has evaluated to what extent this bias occurs. Notably, recent studies have clearly shown that *A. baumannii* is much more than a human pathogen (Rafei et al., 2015; Wilharm et al., 2017; Hernández-González and Castillo-Ramírez, 2020; Mateo-Estrada et al., 2022). These studies point to the fact that this bacterium might dwell in other species (and sources) beyond the humans and hospital environments (Castillo-Ramírez, 2022).

Unsupervised learning and natural language processing (NLP) are two areas of Artificial Intelligence (AI) that have been instrumental in gaining better biological insights considering large collections of biomedical literature. NLP encompasses several techniques to generate and understand natural language (Manning et al., 2009). An unsupervised learning approach receives unlabelled data as input and learns a model to describe the relationships in the data. The classical technique of unsupervised learning is clustering, which discovers groups of similar examples from the data using a similarity function (Bishop and Nasrabadi, 2006; Aggarwal and Zhai, 2012). Some previous studies have used clustering approaches to extract knowledge from biomedical articles but without any attempt to describe an organism or disease (Boyack et al., 2011; Renganathan, 2017). On the other hand, descriptions of diseases using biomedical literature have been accomplished by applying topic modelling (Zhao et al., 2020; Dastani and Danesh, 2021; Dastani et al., 2022). Topic modelling is a statistical NLP analysis to identify representative terms (technically called *topics*) in document collections, where these representative terms are defined by a probability distribution of words (Dastani et al., 2022). In this work, we used clustering to discover themes, and we performed topic modelling on the clusters to assist clustering interpretation. In particular, we have used AI-based approaches (machine learning and NLP) to better understand some aspects of the transcriptional regulation of one of the most studied organisms (*Escherichia coli*) and one of the main pathogens infecting humans and animals worldwide, namely *Salmonella enterica* serovar Typhimurium (Méndez-Cruz et al., 2017, 2020). By training an AI model for text summarisation, we were able to describe the properties of 177 bacterial transcription factors (TFs) of *E. coli* using 5,961 scientific articles, and 185 TFs of *S. enterica* using 3,498 articles. Our results demonstrated the feasibility of automatically extracting knowledge to describe bacterial TFs from large collections of biomedical literature.

To date, there has not been any approach trying to implement clustering and NLP to appreciate the diversity of research themes regarding *A. baumannii*. Here, we use these approaches to define and characterise the research trends (and thus research efforts) about this important human pathogen. We assumed that each cluster represents a research theme (theme cluster) and the size of the cluster (number of publications) quantifies the attention/effort put into that theme. Our analysis shows that clear bias exists in the research efforts on this pathogen. We think our approach will be a reference point for similar studies focused on other ESKAPE pathogens.

## 2 Materials and methods

**Figure 1:**
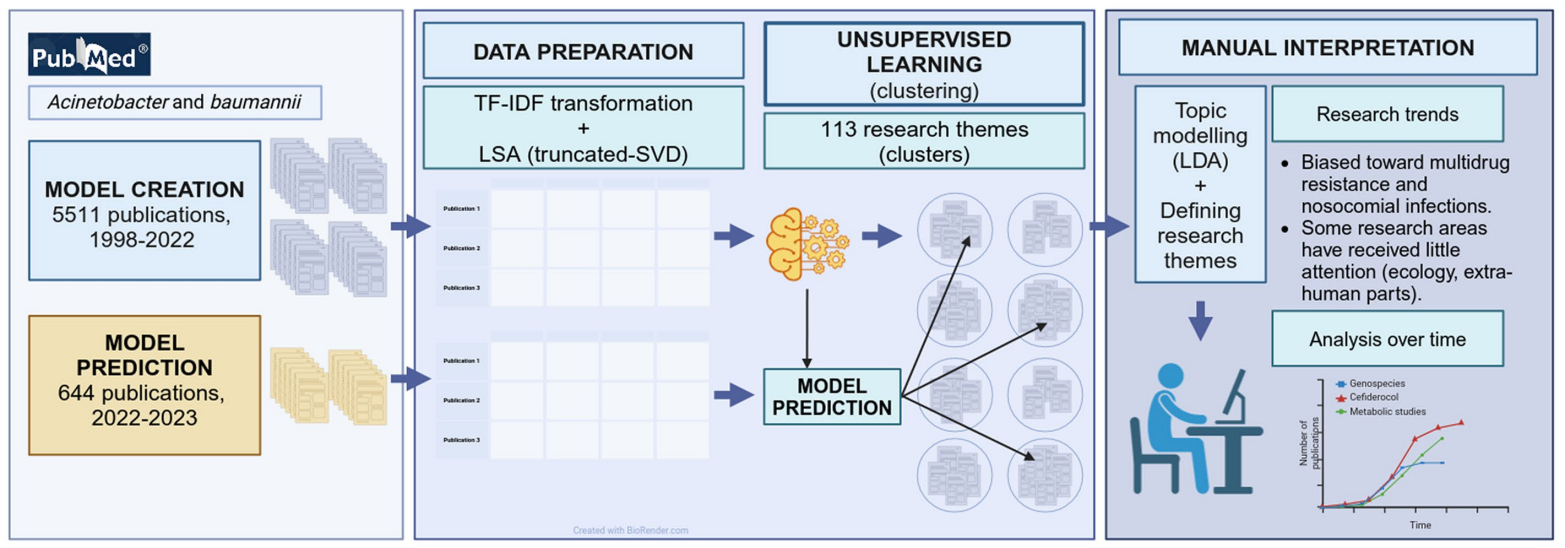
Study workflow depicting data collection and preparation, unsupervised learning (clustering) and manual interpretation.

A graphical study workflow including data collection and preparation, unsupervised learning (clustering) and manual interpretation is depicted in Figure 1. We describe these stages of our study in the following sections.

### 2.1 Study design and data preparation

In this study, 6,155 publications containing the terms *Acinetobacter* and *baumannii* in the title were recovered from the PubMed system as of May 23rd, 2023 (details in Supplementary material). From these publications, we utilised 5,511 published until May 12th, 2022 to discover research trends by obtaining a descriptive model with unsupervised learning. The 5,511 publications were published in 851 different journals of which 108 journals had 10 or more articles (Supplementary Figure S1). The oldest article was from 1988 and the collection covered all years from then to 2022 (Supplementary Figure S2). The remaining 644 publications published from May 13th, 2022 to May 23rd, 2023 were used for model inference (prediction of the clusters), to show the capability of our model to be updated as time goes by. The unsupervised learning analysis using the 5,511 publications comprised two steps: (1) text pre-processing to obtain a numerical matrix representation of the publications, and (2) clustering analysis to learn the descriptive model using this matrix as input.

We performed text pre-processing using the title and the abstract of the publications as both attributes are commonly used for text clustering (Boyack et al., 2011; Ozaydin et al., 2017; Kim and Delen, 2018; Islamaj et al., 2020). The title and the abstract for each publication were concatenated and then tokenized and lemmatized. The tokenization task consists of dividing an input text into words, punctuation, numbers, and symbols, all of which are called tokens. In the lemmatization task, each inflected word of an input text is substituted with a normalised (dictionary) form, named lemma. The inflected verbs are substituted by the infinitive form, for example, studying, studied and studies by study; and the inflected nouns are substituted by the singular form, for example, strains by strain. This step allows a better capture of the differences and similarities among publications. After these two tasks, each publication was represented by the sequence of lemmas, punctuations, numbers and symbols (all will be referred to as tokens) of the title and the abstract. We transformed each publication into a numeric vector so that the set of vectors constitutes a matrix representation of the collection. In this representation, all unique tokens (lemmas, punctuation, number, and symbols) of all publications were the columns of the matrix, and the cells were filled with a weighted frequency of each token in each publication using the *tf-idf weighting scheme* (Sparck Jones, 1972; Manning et al., 2009; Weiss et al., 2010).

We performed a dimensionality reduction of the tf-idf matrix using a truncated singular value decomposition method (truncated-SVD) as this matrix was high-dimensional with approximately 10,000 columns. The truncated-SVD is a kind of matrix decomposition that, when it is applied to text data, is usually called Latent Semantic Analysis (LSA) or Latent Semantic Indexing (LSI). LSA has been considered for a long time as an approach to organise a document into a “semantic structure,” and it has been observed that LSA outperformed using keywords to describe documents (Dumais et al., 1988; Deerwester et al., 1990). This method kept most of the variance in the data by identifying a linear subspace in the columns (features) of a tf-idf matrix that captured semantic relations among tokens, such as synonymy and polysemy (Papadimitriou et al., 1998). The output of the truncated-SVD reduction was a low-dimensional representation of the tf-idf matrix. With this new representation, a clustering analysis improves (Alpaydin, 2020), because this is a better representation of the publications that includes only the principal eigenvectors (Manning et al., 2009). Moreover, dimensionality reduction before the clustering analysis is useful for Euclidean distances not to become inflated (Aggarwal et al., 2001). The number of columns of the reduced matrix is generally established in the range of the low hundreds, so we reduced the tf-idf matrix to 300 dimensions. Tokenization and lemmatization were done with the Stanza NLP library (Qi et al., 2020). Tf-idf matrix creation and dimensionality reduction were performed with the Python scikit-learn library (Pedregosa et al., 2011).

### 2.2 Unsupervised learning

We created a descriptive model using a clustering analysis of the 5,511 publications. The clustering analysis is an unsupervised learning approach to find the underlying organisation of a collection of examples by grouping similar ones into disjoint clusters considering a high intraclass similarity and a low interclass similarity (Igual and Seguí, 2017). We considered that each cluster may represent a theme of research interest.

We employed the k-means algorithm (Lloyd, 1982), which is one of the most popular hard partition algorithms used in science and industry (Berkhin, 2006). This algorithm divided the collection of publications, represented by the reduced tf-idf matrix, into *k* disjoint clusters, each described by the mean of the publications in the cluster, called *cluster centroid* (Igual and Seguí, 2017). To obtain consistent clusters, we employed two strategies. First, to mitigate the problem of local minima of the k-means algorithm, we utilised the approach *greedy k-means++* to select initial cluster centroids (Arthur and Vassilvitskii, 2007). This approach selects the best initial cluster centroids among several trials at sampling on a probability distribution. The selected initial cluster centroids tend to be distant from each other, leading to better results than random initialization. The second strategy was to iterate up to 300 times each run of the algorithm.

The k-means algorithm requires the *k* number of clusters as input (algorithm in Supplementary material). To select the best *k*, we evaluated the quality of different clustering analyses with values of *k* from 50 to 500 using the Silhouette coefficient (Rousseeuw, 1987). Calculating this coefficient is an unsupervised approach for clustering evaluation, that is when the collection of examples is not required to be previously manually assigned to labels. The Silhouette coefficient was calculated for each publication, so the average for all publications of a given cluster defined its quality. This coefficient goes from −1 to 1, and a value close to 1 means a compact, dense and well-separated cluster (Igual and Seguí, 2017). The average of the Silhouette coefficient of all clusters gave the quality of the clustering. Silhouette coefficient calculation and k-means clustering analyses were achieved using the scikit-learn library (Pedregosa et al., 2011).

The next action was the interpretation of each cluster in the final best clustering. This was performed in two steps: (1) we performed a topic modelling analysis of each cluster to automatically obtain a set of representative terms describing the cluster, we also obtained for each cluster the 10 top terms from the tf-idf matrix that described the cluster centroids, and (2) we manually curated the clusters by assigning a label (short phrase) describing the theme of the cluster considering the terms from the topic modelling and the centroids.

Topic modelling is a statistical approach to discovering thematic information in a document collection (Blei, 2012; Albalawi et al., 2020). This thematic information is represented by abstract topics, which are a set of representative terms (lemmas) that probably co-occur together. The name of the abstract topic must be manually assigned, as the topic modelling only suggests a list of representative terms. For example, a topic modelling performed over 17,000 articles from the journal *Science* inferred the abstract topic *Genetics* characterised by the representative terms *human, genome, dna, genetic, genes, sequence, gene, molecular, sequencing, map, information, genetics, mapping, project, sequences* (Blei, 2012); note that the name of the abstract topic, *Genetics*, was manually assigned by the authors, as the topic modelling only suggests the list of representative terms. We discovered abstract topics with the Latent Dirichlet Allocation (LDA) method, which is one of the most common topic modelling approaches (Pritchard et al., 2000; Blei et al., 2003).

As the topic modelling is an unsupervised approach, the best number of topics for all clusters of publications was not automatically obtained by the LDA method, so we had to find this number. We performed an LDA analysis from one to ten topics over the lemmas and ordered pairs of lemmas of the publications of each cluster and we recorded the coherence score for each of the 10 LDA analyses. The coherence score measures if the representative terms of a discovered abstract topic probably co-occur together (Mimno et al., 2011); this score was calculated using the Mallet library (McCallum, 2002). Then, we calculated the average of the coherence score of all clusters for the 10 LDA analyses. We selected the number of topics with the lowest negative value of the average coherence score, as this value indicates that the representative terms of the discovered abstract topic do not co-occur most often and may better describe the theme of the cluster (terms co-occurring most often will tend to be multi-word terms instead of representative terms). Selecting the best number of topics by using the average of the coherence score of all clusters results in all the clusters being described by the same number of topics.

In addition, we tried to elucidate if the representative terms obtained with the LDA analysis and from the centroids may be automatically used as labels of clusters. This was important considering that a manual interpretation of clusters in the clustering of large collections of publications is demanding and time-consuming, therefore assigning automatically a label to the clusters may be truly beneficial. Pursuing this goal, a PhD student (VME) who conducts research on *A. baumannii* and who was not involved in the clustering and did not know the clustering results, was given a 10% of randomly selected clusters and was asked to assign a short phrase describing the content of each cluster by reviewing only the title and, if necessary, the abstract of the publications.

## 3 Results

### 3.1 Clustering analysis and topic modelling

**Figure 2:**
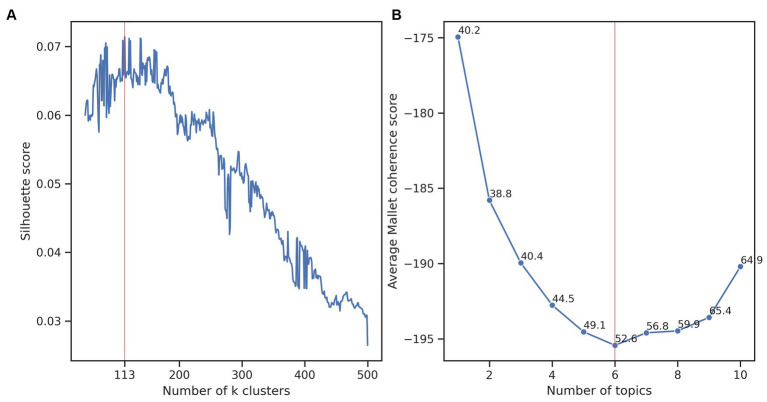
Finding the best clustering and topic modelling. **(A)** Average silhouette score considering 50 to 500 clusters. The highest score (denoted with the red vertical line) was 0.071 and was obtained with 113 clusters. **(B)** The average Mallet coherence score of the LDA topic modelling considering 1 to 10 abstract topics for the 113 clusters. The lowest score (red vertical line) was −195.42 obtained with six topics. We included for each number of topics, the Mallet coherence standard deviation.

A workflow of our approach is presented in Figure 1. To discover trends in the research of *A. baumannii*, we interpreted and analysed the clusters in the best clustering obtained for the 5,511 publications. Clustering analysis was done with the k-means algorithm using a reduced matrix representation of the publications. We selected the best clustering using the highest silhouette score. The best clustering was formed from 113 clusters, with an average silhouette score of 0.071 over 450 runs from 50 to 500 clusters (Figure 2A). This score indicated that the clustering contained well-defined clusters, but also showed the hard task of partitioning these publications. To interpret the best clustering, we manually inspected each cluster to assign a label (a short phrase) describing the theme of the cluster. As mentioned earlier, we also calculated the average of the coherence score of all clusters for one to ten LDA analyses to select the best number of topics. We found that six topics obtained the lowest average Mallet coherence score of −195.42 (Figure 2B). Then, as each of the six abstract topics was a set of several representative terms, we had to decide on some representative terms to describe each cluster. Because we manually assigned the label to each cluster, we considered that 12 representative terms (the first two representative terms of each of the six abstract topics) and the ten terms associated with the cluster centroids were appropriate to perform the manual task (a total of 22 terms). Generally, 5 to 20 terms are typically examined (Crain et al., 2012), but a more elaborated strategy to select this number of terms will be considered in future work.

Importantly, we conducted manual curation of the clusters. We created a clustering table with the following attributes for the 5,511 publications: title, abstract, date of publication, authors, journal ISO abbreviation, 12 LDA terms, and ten terms from cluster centroids (Supplementary Table S1, details in Supplementary material). Taking into consideration these terms, we manually labelled each cluster by assigning a short phrase describing the theme of the cluster and creating a clustering labelling table that was used for downstream analysis and the eventual discovery of research trends. This table included the cluster id (from 0 to 112), the total publications in the cluster, the manual label, the start year (year of the oldest publication), the end year (year of the most recent publication), the LDA terms, and the terms from cluster centroids (Supplementary Table S2, details in Supplementary material). A sample with two of the smallest clusters and two of the largest clusters of this table is shown in Table 1 (another way to visualise the conceptual context of each cluster, i.e., LDA topics and terms from cluster centroids, is shown in Supplementary Table S3). Collectively, these results suggest that the 5,511 publications can be organised in well-defined clusters, that in turn, can be defined by just 6 abstract topics characterised by 12 representative terms (see column *LDA terms* in Table 1), and 10 terms from the cluster centroids.

**Table 1:** Four clusters were selected to illustrate manual clustering interpretation.

Cluster id	Total of publications	Start year	End year	Label	LDA terms	Terms from cluster centroids
15	14	2012	2022	Polymyxin and metabolic/metabolomic studies	Metabolic difference nab fic combination synergistic polymyxins polymyxin mechanism pathway genome-scale flux	Polymyxin combination pathway polymyxins synergistic baumannii acid metabolic kill metabolomics
81	14	2013	2021	Colonies, opacity, translucent, phenotypes	Opaque translucent colony sup mucoid phenotype variant ompr capsule cell switch avt	Variant colony phenotype switch translucent opaque capsule virulence phenotypic avt
11	126	2006	2021	Carbapenem-resistant *A. baumannii*, blaOXA genes and OXA genes	Clone blaoxa imipenem caro isaba like type clonal carbapenemase resistant blaoxa_like pcr	Blaoxa like gene isaba carbapenem resistance strain carbapenemase hospital carry
63	110	1998	2022	Nosocomial, clinical treatments and infections, healthcare	Growth protein south bacterial antimicrobial gram-negative pathogen hospital new cause treatment option	Resistance antimicrobial pathogen hospital multidrug-resistant cause nosocomial resistant healthcare agent

The table includes cluster id, total publications in the cluster, start year (year of the oldest publication), end year (year of the most recent publication), manually assigned label, LDA terms, and terms from cluster centroids. The complete table is available in the Supplementary Table S2, and description in Supplementary material.

### 3.2 Defining research themes

To define research themes, we analysed some features of the clusters included in the cluster labelling table (Supplementary Table S2): the label, the size (number of publications), and the time span of the publications. First, we noted that just five clusters have more than 100 papers. These are clusters 104, 11, 63, 21, and 70. They refer to antibiotic resistance, antibiotic resistance genes, clinical/hospital settings, outbreaks, etc. (see Table 2). In this regard, antibiotic resistance seems to be the overarching theme. This also can be appreciated in the two top journals publishing research about this pathogen: Antimicrobial Agents Chemotherapy and Journal of Antimicrobial Chemotherapy. Between the two of them, they have published over 500 articles about *A. baumannii*. Thus, it seems that the clinical relevance of this pathogen has driven much of the research efforts.

**Table 2:** The five largest clusters.

Cluster id	Total of publications	Label	Start year	End year
104	173	Multidrug-resistant *A. baumannii* and extensively drug-resistant *A. baumannii*	2010	2022
11	126	Carbapenem-resistant *A. baumannii*, blaOXA genes and OXA genes	2006	2021
63	110	Nosocomial, clinical treatments and infections, healthcare	1998	2022
21	108	Intensive care units (ICUs)	1993	2022
70	107	*In vitro* antimicrobial combination and synergy/synergistic	1995	2022

The size of the clusters was defined by the number of publications in it.

We now describe some themes that have received much less attention. Clusters 83 (*Structural studies of O-specific polysaccharide*) and cluster 5 (*Genospecies*) have just 16 and 18 publications, respectively (see Table 3, first and second rows). We chose the two clusters as they represent very different research areas. For instance, whereas Cluster 83 belongs to polysaccharide research, “Genospecies” pertains to taxonomy and systematics. The last publications from these clusters were published in 2018 and 2015, respectively. These findings may indicate that these themes have been overlooked in the last few years or are defunct themes. To support this observation, we checked if these themes were present in recent publications of other clusters by searching the words of the label in the title and abstract of all clusters. In the case of studies related to *Genospecies* (cluster 5), the word *genospecies* is mentioned after 2018 only in one publication (Leung et al., 2019) from 2019 in cluster 69. No additional mentions of this word were found. The case of the theme *Structural studies of O-specific polysaccharide* (cluster 83) is more extreme. For instance, the words like *O-specific*, *O18 antigen*, *O-7 antigen*, and *O10 antigen* appear exclusively in cluster 83. Importantly, some research themes are bound to dwindle given the progress of science and technology. *Genospecies* is a good example. Initially, many *Acinetobacter* species were provisionally classified as genospecies. However, due to further studies using more advanced techniques most of those “genospecies” have now been properly characterised and now they have a regular species name. Therefore, it seems that our approach can identify themes that, despite receiving attention some time ago, have declined over the last few years.

**Table 3:** New and defunct research themes.

Cluster id	Total of publications	Label	Start year	End year
83	16	Structural studies of O-specific polysaccharide	1994	2015
5	18	Genospecies	1989	2018
**78**	19	Cefiderocol against *A. baumannii*	2019	2022
**84**	33	*In vitro* and *in vivo* studies/activity/efficacy	2017	2022

Clusters 5 and 83 show themes that have lost attention in research as the end year of these clusters (year of the most recent publication) was 2015 and 2018, respectively. The themes of these clusters were not mentioned in recent publications. On the contrary, clusters 84 and 78 (in bold) show themes that recently emerged as their first (oldest) publications were published in 2017 and 2019, respectively.

Contrary to the previous trend, to identify themes that may have gained interest recently, we reviewed clusters with publications published between 2017 and 2022 (Table 3, last two rows, id clusters in bold). A salient case is cluster 78, which describes publications on Cefiderocol and *A. baumannii*. Of note, this antibiotic (Cefiderocol) is used against multidrug-resistant Gram-negative bacterial infections, which was very recently approved in the United States (November 2019) and in the European Union (April 2020). Considering cluster 78, the first studies were published in 2019. To be sure that no previous publications on Cefiderocol were in the rest of the clusters, we searched the word *cefiderocol* in all clusters. Notably, no mention of this word was found in either the title or the abstract of articles published before 2019, which confirmed that studies of this antibiotic are of recent interest. In agreement with our findings, papers about Cefiderocol started to appear in 2016 and 2017 in PubMed but were about other non-*A. baumannnii* pathogens.

We also reviewed the 10 smallest clusters (Table 4). Cluster 81, with just 14 publications, gathered studies of opaque or/and translucent colonies. Notably, the word *opacity* appeared exclusively in cluster 81 and the word opaque is only mentioned in cluster 41 in one publication (Ahmad et al., 2019). Whereas, the word *translucent* is just mentioned in three abstracts outside cluster 81. These findings show that a few studies on this theme have emerged in the last decade.

**Table 4:** The 10 smallest clusters by number of publications.

Cluster id	Total of publications	Label	Start year	End year
81	14	Colonies, opacity, translucent, phenotypes	2013	2021
15	14	Polymyxin and metabolic/metabolomic studies	2012	2022
67	15	Pan-drug-resistant *A. baumannii* in Taiwan	2002	2021
83	16	Structural studies of O-specific polysaccharide	1994	2015
74	17	Secretion system, mainly vgrG/VgrG	2013	2022
5	18	Genospecies	1989	2018
78	19	Cefiderocol against *A. baumannii*	2019	2022
82	19	Combination of antibiotics against *A. baumannii*; especially, colistin, rifampicin and imipenem	2006	2020
10	20	*A. baumannii* in human body louse, meat, falcon, milk, faecal samples from non-hospitalised individuals	2004	2022
85	20	Outer membrane proteins OMP, OmpA, OMPs	1996	2022

Cluster 10 gathered studies of the detection of *A. baumannii* in non-human sources (“extra-human parts”). This cluster contains a couple of publications about isolates of *A. baumannii* found in animals. It also has studies about *A. baumannii* from meat, except for one study assigned to cluster 104 (Elbehiry et al., 2021). It even gathers publications about fecal/faecal samples from non-hospitalised individuals, except two from clusters 103 and 11. Cluster 10 clearly shows investigations on non-human, non-clinical samples yet it also highlights the scarcity of such studies.

### 3.3 Studying research themes over time

**Figure 3:**
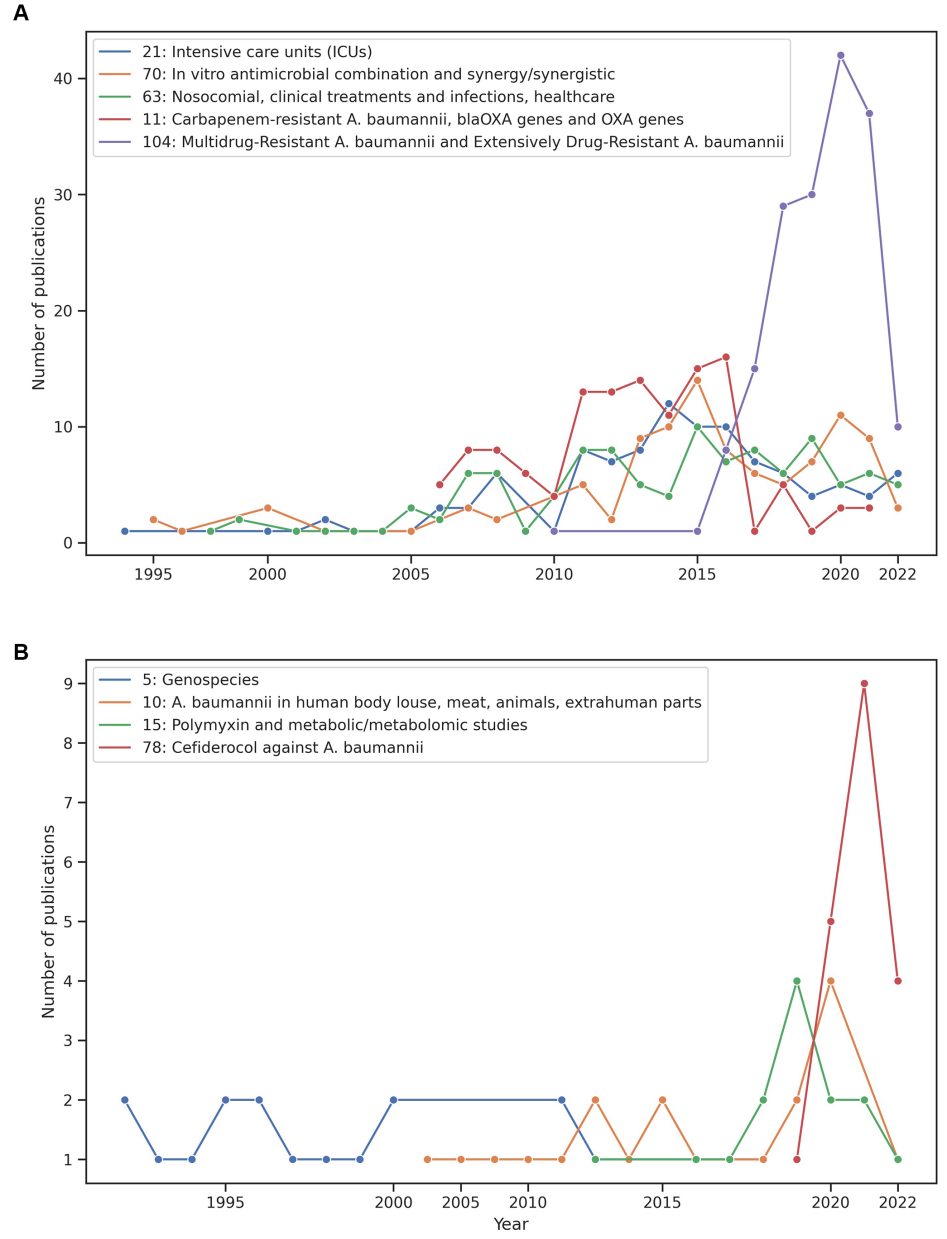
Temporal trends of some clusters. Comparison between some of the relevant smallest clusters **(B)** and the largest clusters **(A)**. The year 2022 is covered until May.

Our approach can also be very useful to analyse the research themes over time. Figure 3 shows the time trends of some of the smallest and largest clusters. The emergence and decline of some of these themes can be easily appreciated in this figure. We comment on two cases relevant to antimicrobial therapy and clinical microbiology. For instance, we previously mentioned the case of cluster 78 about Cefiderocol and Figure 3B (red line) shows this theme emerged in 2019 and has an increased number of published papers since then. We also noted the hegemony and the rapid increase of studies of *A. baumannii* as a multidrug and extensively drug-resistant bacterium (see Figure 3A). Figure 3A shows those clusters that have received the most attention (research effort) based on the number of publications per cluster. Here there are cases very important not only from a clinical microbiology point of view but also from an antimicrobial therapy angle. For instance, the purple line shows MDR and XDR *A. baumannii* (a very relevant and current theme in clinical microbiology) over time. Whereas the red line denotes the Carbapenem-resistance *A. baumannii* theme across time. Of note, in Figure 3, the year 2022 is covered until May, so the dramatic decrease in publications might not be real for many clusters. Taken together, these results show that our approach can be very useful not only to define but also to analyse the research trends over time. Finally, we noted that some research themes have been completely neglected regarding the research on *A. baumannii*. For instance, none of the clusters had the word *ecology* in its label and this word was found only in 7 articles in the clustering table with the 5,511 articles. On closer inspection of these 7 articles, we noted that most of them are not ecological studies. Of note, there are other ways to analyse temporal trends and in future studies we will use these alternative techniques. For example, dynamic topic modelling technique (Blei and Lafferty, 2006) or techniques to discover clusters with text streams, such as the Online Spherical k-Means Algorithm (Zhong, 2005; Aggarwal and Zhai, 2012).

### 3.4 Assessing cluster labelling and model predictions

To evaluate if terms obtained with the LDA analysis and from the centroids can be automatically used as labels of clusters, we conducted the following procedure. We obtained the number of LDA terms, terms from centroids, and the overlapped terms between both, which appeared in the manually assigned short phrase by a PhD student (VME) of the 10% percent of randomly selected clusters (5, 13, 22, 24, 43, 49, 77, 78, 81, 87, 91, 109). The short phrases were lemmatized, changed to lowercase, and stop words, punctuation and symbols were removed before calculation. We found that, on average, in the short phrase appeared 21.5% of the LDA terms, 35% of the terms from centroids, and 50% of the overlapped terms between both. For five clusters, the overlapped terms matched with more than 65% of words in the short phrase (cluster 13: 100%, cluster 49: 83%, cluster 43: 80%, cluster 5: 75%, cluster 91: 67%), which was a relevant finding. For six clusters, the overlapped terms matched between 20% and 42%. These results show that assigning the overlapped terms as labels for clusters may be a promising approach to automatically describe them, being relevant for large collections. Details of these results are shown in Supplementary material, Supplementary Figure S3, and Supplementary Table S4.

Finally, we used the model produced by the clustering to predict the cluster for the 644 publications published between May 13th, 2022 and May 23rd, 2023). These publications were assigned to 100 different clusters, with a mean of 6.4 and a median of 5 per cluster (Supplementary material and Supplementary Figure S4). As expected, the cluster that attracted more publications was cluster 104 (*Multidrug-Resistant A. baumannii and Extensively Drug-Resistant A. baumannii*), which was augmented in 64 publications. A relevant finding was that cluster 78 (*Cefiderocol against A. baumannii*) increased by 12 publications, this increment was in the highest 15 increments, confirming that this is an active theme in studies of *A. baumannii*. These predictions show the capability of our model to be updated as time goes by, allowing a continuous vigilance of research trends. A table with predictions for the 644 publications is shown in Supplementary Table S5.

## 4 Discussion

An understanding of the research trends of *A. baumannii* and other ESKAPE pathogens is relevant not only from a basic research point of view but also from a translational one. To the best of our knowledge, this is the first time that unsupervised learning and NLP have been employed to discover the research trends of this important pathogen. Notably, compared with systematic reviews, our approach is much more inclusive in terms of academic themes. Most of the systematic reviews just focus on the issues of antibiotic resistance or treatments against *A. baumannii* (Lemos et al., 2014; Zhihui Jiang and Li, 2018; Mei et al., 2019; Lyu et al., 2020). On the contrary, our query was very general just specifying the name of the species and, thus, allowing us to recover research studies on many different scientific areas. Thus, our approach has “no bias” in the sense that it is conducted without any particular perspective in mind, i.e., clinical microbiology, antimicrobial agents and chemotherapy, or biochemistry. Additionally, the data set here gathered is very extensive, as it can be appreciated not only by the number of publications and different journals included but also by the time span covered by the publications. Of note, rather than provide an exhaustive analysis of the research trends, the main goal of this study was to show that our strategy is able to define the research trends for a given pathogen or infectious disease.

In this study, we used the k-means algorithm to cluster articles instead of classical topic modelling, another commonly utilised strategy for clustering. Clearly, both approaches have advantages and drawbacks. However, processing biomedical literature imposes specific challenges, for example, the high-dimensionality representations and redundancy of words in biomedical literature have a negative impact on topic modelling (Rashid et al., 2019). For our clustering analysis, this impact was minimised by applying the truncated-SVD dimensionality reduction technique. One of the main disadvantages of classical topic modelling is the run-time to model the complete set of publications, as it is a generative probabilistic approach. A partitioner algorithm, such as k-means, does not have this limitation and may process a large number of publications in a shorter run-time. It is important to mention that there is no conclusive evidence that classical topic modelling techniques, such as LDA, surpass the clustering algorithms. Furthermore, using clustering algorithms to analyse biomedical literature has been done for a long time (Andreopoulos et al., 2009). For example, there have been studies conducting themes (topics) discovery using hybrid inverse document frequency and fuzzy k-means instead of classical topic modelling (LDA and LSA). There is even a study demonstrating that the performance of the clustering technique was better than the LDA (Rashid et al., 2019). Recently, there have been studies suggesting that using clustering with pre-trained word embeddings is a better strategy in terms of runtime and computational complexity than classical topic modelling approaches (Sia et al., 2020). Another study showed that for health-related short texts (tweets and emails) clustering performed better when evaluated by external indices, while classical topic modelling performed better when evaluated by internal indices (Lossio-Ventura et al., 2021). Moreover, there is work to integrate clustering and topic modelling to combine the advantages of each approach (Xie and Xing, 2013; Alhawarat and Hegazi, 2018).

Our strategy may have some limitations. First, we restricted our information recompilation just to PubMed, yet we needed to do this for practical reasons. Nonetheless, we think the publications found in PubMed are a fair representation of most of the worldwide publications concerning *A. baumannii*. Secondly, we analysed the title and abstract (titles+abstracts) of the publications, but not the full-text article. Using abstracts versus full-text articles has been a long-term theme of discussion in the NLP field as both have specific properties (Cohen et al., 2010). It has been pointed out that using full-text articles has challenges, such as recognition of non-ASCII characters, figures, tables and errors in converting PDF to textual format (Cohen et al., 2010). Also, it has been observed that abstracts have the most important (novel) findings, while the introduction of full-text articles contains existing knowledge (Westergaard et al., 2018). We consider that the decision of using full-text or only titles+abstracts may be guided by the objective of the study, in our case, finding research trends (themes). For example, it has been mentioned that for recovering biomedical and clinical relations (gene-protein, treatment-disease) using full text is the better strategy (Blaschke and Valencia, 2001; Blake, 2010; Westergaard et al., 2018). However, studies on information retrieval found that using full text does not outperform using abstracts (Lin, 2009). Also, several works aiming at discovering research trends in medical articles have used only abstracts (Ozaydin et al., 2017; Kim and Delen, 2018). Other studies in the literature of diseases have used only titles, abstracts, and keywords, such as those for COVID-19 (Dastani and Danesh, 2021) and for Brucellosis (Dastani et al., 2022). Another study only employed the abstract and conclusion of the articles to analyse factors in documents about global renewable energy (Kumar and Ng, 2022). Importantly, another concern is that using full-text articles implies having open access rights to publications, which will be a drawback for analysing large collections of publications; using titles+abstracts does not have this limitation. Furthermore, some studies, instead of processing the whole manuscript, prefer to include the MeSH terms (Boyack et al., 2011; Islamaj et al., 2020). We will add the use of MeSH terms in future analyses. Finally, from the computational point of view, it has been stressed that one challenge for text clustering is the sparsity of the high dimensional text representations that enter into clustering algorithms, because only a few 100 words are contained in each document, so the selection of the best document description is necessary (Aggarwal and Zhai, 2012). In biomedical literature, full text may include tables, references and experimental descriptions that may increase the vocabulary present in the article and therefore increase the sparsity of the representation of document collections; this was another reason to employ only titles and abstracts in our study.

A few limitations come from the drawbacks of the k-means algorithm (Manning et al., 2009; Ikotun et al., 2023). This algorithm depends on the initialization of cluster centroids, nonetheless, we addressed this issue by selecting the greedy k-means++ initialization approach. Also, this algorithm may be affected by outliers generating *singleton clusters*, that is, clusters with only one document. We did not obtain any singleton cluster in our study, and our manual inspection of the smallest clusters revealed consistent results (see, for example, clusters 81, 83, 5). Thus, we consider that outliers did not affect our clustering analysis. In addition, clusters in this algorithm are assumed to be spheres, this means that the algorithm assumes that the publications of the same theme are distributed in a spherical area, which may be not true. A possible effect of this assumption may be that the algorithm splits a large non-spherical cluster into some spherical clusters, but this effect does not have to be unfavourable, as the resulting spherical clusters may represent subthemes. Moreover, knowing beforehand the underlying structure of the data is not trivial, and this structure depends on how the data is pre-processed. Despite these limitations, our study is worthy as it provides a general view of the research trends (and by implication research efforts) of *A. baumannii*.

This study has some very important implications, besides basic research. First, the strategy followed here can be applied to other very important bacterial and non-bacterial pathogens for which vast amounts of information are available. For instance, for *S. aureus* using the query “Staphylococcus AND aureus,” as of 28th of June, 2023, there were 148, 078 PubMed entries; clearly, a systematic review cannot deal with this vast amount of information. Nonetheless, our strategy can be implemented to have a global view of the research trends about *S. aureus*. However, in this case, manual curation of the clusters might not be feasible. Secondly, the research themes found (the clusters) on *A. baumannii* can be very useful to funding research agencies all over the world. Along these lines, funding agencies can enhance efforts (and even allocate more money) to those neglected research themes of *A. baumannii*. Some of these neglected areas could provide the scientific basis for important actions such as infection control, target prevention and alternative treatment therapies. Opening specific calls focusing on these understudied aspects would be very beneficial to fully understand the whole biology of this species. Also, the list of the research themes is bound to be important to researchers working on this species, as they would better understand which scientific areas have been most studied and which have been neglected. Along these lines, this study was very helpful for one of us (SCR), who recently wrote a comment on the relevance of non-human populations of *A. baumannii* (Castillo-Ramírez, 2023). Finally, our results can be very useful for researchers from many different scientific areas as they can have a big picture of the knowledge generated for *A. baumannii* thus far.

In conclusion, our approach provides an overview of the research trends of *A. baumannii*. Clearly, scientific areas relevant to human health have been densely studied yet some basic biological aspects of this pathogen have been neglected. We anticipate that similar studies will be conducted on other important human pathogens.

## Data availability statement

The original contributions presented in the study are included in the article/Supplementary material, further inquiries can be directed to the corresponding authors.

## Author contributions

C-FM-C: Conceptualization, Funding acquisition, Investigation, Methodology, Software, Supervision, Writing – original draft, Writing – review & editing. JR-H: Formal analysis, Software, Writing – review & editing. AV-V: Formal analysis, Software, Writing – review & editing. VM-E: Data curation, Formal analysis, Writing – review & editing. SC-R: Conceptualization, Investigation, Methodology, Supervision, Writing – original draft, Writing – review & editing.
