# Supplementary material for: Unsupervised learning and natural language processing highlight research trends in a superbug
Source: Front Artif Intell. 2024 Mar 21;7:1336071. doi: 10.3389/frai.2024.1336071 (PMC10991725; doi:10.3389/frai.2024.1336071)
Supplement: Supplementary file 1 [file Presentation_1.pdf]

Supplementary Material

1 Supplementary Methods

1.1 Procedure for gathering publications

To gather the publications for training the unsupervised clustering model, we searched on the PubMed system (https://pubmed.ncbi.nlm.nih.gov/) on May 12th, 2022 for documents containing the terms *Acinetobacter* and *baumannii* in the title with the query: “Acinetobacter[TI] AND baumannii[TI]”. A total of 6,035 results were obtained. We saved the list of PubMed identifiers (PMIDs) using the PMID format of the *Save* button. Using the Bio.Entrez package from biopython API (https://biopython.org/docs/1.75/api/Bio.Entrez.html), we downloaded the following attributes for each article: PMID, title, abstract, publication date, authors and journal ISO abbreviation. We retrieved a final article collection with 5,511 publications with all attributes (523 did not have an abstract and one PMID was not found by the Bio.Entrez package). To recover the publications used for model inference (prediction of clusters) from May 13th, 2022 to May 23rd, 2023 , we performed the same steps in the PubMed system on May 23rd, 2023 with the following query: “((Acinetobacter[Title]) AND (baumannii[Title])) AND (("2022/05/13"[Date - Publication] : "2023/05/23"[Date - Publication]))”. The PubMed system retrieved 663 publications from which we considered 644, as 19 did not have an abstract. A total of 6,155 publications were included in the complete study.

1.2 Description of gathered publications

The 5,511 publications employed to train the unsupervised model were published in 851 different journals from which 108 journals had 10 or more articles (Supplementary Figure S1). The oldest publication was from 1988 and this collection covered all years from then to 2022 (Supplementary Figure S2).

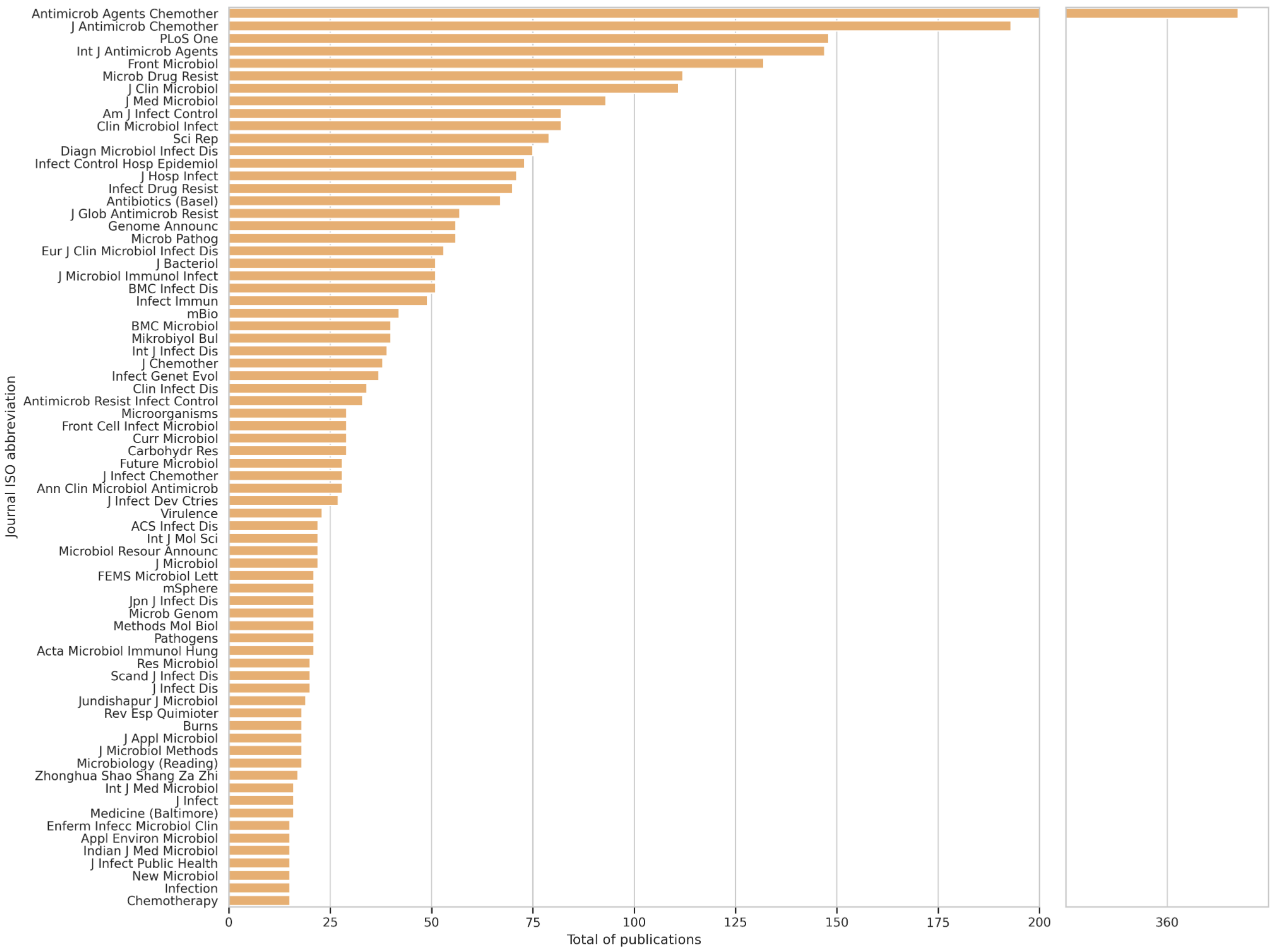

Supplementary Figure S1. Total of publications per journal. We only show journals with 10 or more publications.

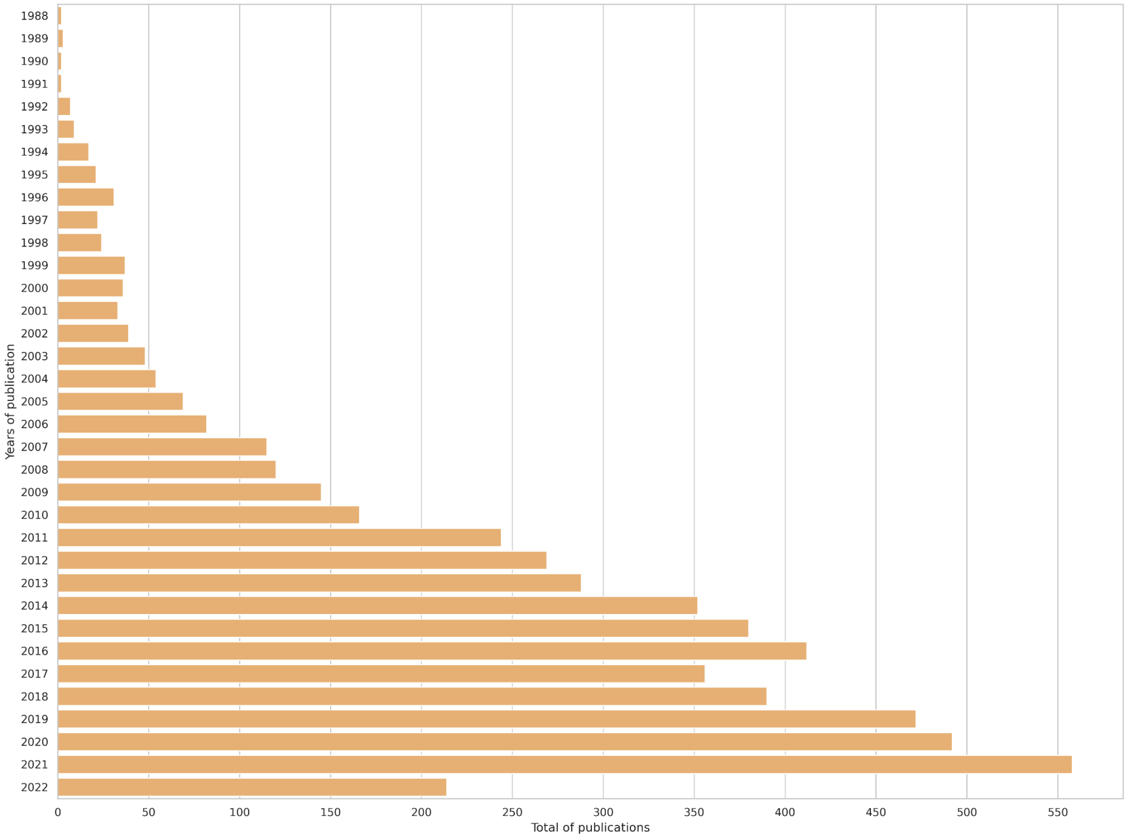

Supplementary Figure S2. Total of publications per year. The year 2022 included publications until May 12th.

1.3 The K-means algorithm

The K-means algorithm may be summarised in the following procedure:

1. Initialization of the k cluster centroids.
2. Decision of the membership of examples by assigning them to the nearest cluster centroid.
3. Re-estimation of the k cluster centroids assuming a correct decision of membership done in the previous step. The new centroids are created by taking the mean value of all of the examples assigned to a centroid.
4. If no example changes its membership from the last iteration, the procedure ends. Otherwise, continue from step 2.

To mitigate the problem of local minima of the K-means algorithm, we utilised the approach *greedy k-means++* to select initial cluster centroids (Arthur & Vassilvitskii, 2007). This approach selects the best initial cluster centroids among several trials at sampling initial cluster centroids based on a probability distribution. The selected initial cluster centroids tend to be distant from each other, leading to better results than random initialization.

2 Supplementary Results

2.1 Clustering analysis and topic modelling

2.1.1 Supplementary Table S1. Clustering table

The clustering table (Table_S1.xlsx) contains the following attributes of the 5,511 publications: cluster id, title, abstract, date of publication, authors, journal ISO abbreviation, 12 LDA terms, and 10 terms from cluster centroid. This table was created joining the results from the clustering analysis and the topic modelling with the Mallet LDA approach. This table was used for clustering interpretation by manually assigning a label to each cluster taking into consideration the LDA terms and the terms from cluster centroids. The table is sorted by cluster id.

Column description

- **Cluster id**: the cluster id assigned by the K-means algorithm, it goes from 0 to 112 for the 113 clusters.
- **PMID**: the PubMed identifier of the publication, obtained from the PubMed system.
- **Title**: the title of the publication, obtained from the PubMed system.
- **Abstract**: the abstract of the publication, obtained from the PubMed system.
- **Date of publication**: the year or year/month of publication, obtained from the PubMed system.
- **Authors**: the authors of the publication, obtained from the PubMed system.
- **Journal ISO abbreviation**: the journal ISO abbreviation of publication, obtained from the PubMed system.
- **LDA terms**: the 12 terms obtained from topic modelling analysis of the publications of the cluster.
- **Terms from cluster centroid**: the ten terms obtained from the cluster centroid calculated by the K-means algorithm.

The table can be downloaded from:

- https://github.com/laigen-unam/research-trends-ab
- https://drive.google.com/drive/folders/18T6HwB9wdKVAn7VKs0nwUXKKvW9euD3K?usp=sharing

2.1.2 Supplementary Table S2. Cluster labelling

The cluster labelling table (Table_S2.xlsx) was the result of interpretation of each cluster. This table includes the following attributes: cluster id, total of publications in the cluster, manually assigned label, start year (year of the oldest publication), end year (year of the most recent publication), LDA terms, and terms from cluster centroids. This is sorted by cluster id.

Column description

- **Cluster id**: the cluster id assigned by the K-means algorithm, it goes from 0 to 112 for the 113 clusters.
- **Total of publications**: the total of publications grouped in the cluster by the K-means algorithm.

- **Label**: the short phrase describing the theme of the cluster. This was manually assigned using the LDA terms and the terms from cluster centroids.
- **Start year**: the year of the oldest publication within the cluster.
- **End year**: the year of the most recent publication within the cluster.
- **LDA terms**: the 12 terms obtained from the topic modelling analysis of the publications of each cluster.
- **Terms from cluster centroid**: the ten terms obtained from the cluster centroid calculated by the K-means algorithm.

Table can be downloaded from:

- https://github.com/laigen-unam/research-trends-ab
- https://drive.google.com/drive/folders/18T6HwB9wdKVAn7VKs0nwUXKKvW9euD3K?usp=sharing

2.1.3 Supplementary Table S3. Four selected clusters to illustrate manual clustering interpretation. LDA terms and Terms from cluster centroids era depicted as word clouds where colour gradient and font size have no meaning, both are just visual aids.

Cluster id	Total of publications	Start year	End year	Label	LDA terms	Terms from cluster centroids
15	14	2012	2022	Polymyxin and metabolic/metabolomic studies	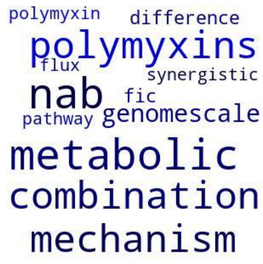	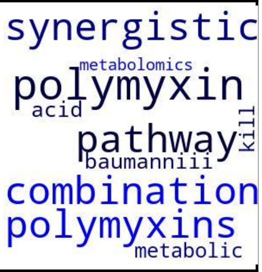
81	14	2013	2021	Colonies, opacity, translucent, phenotypes	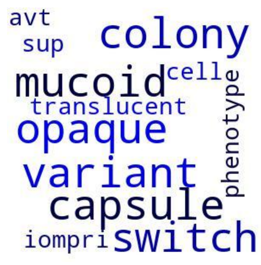	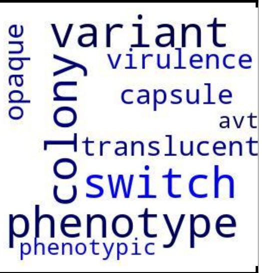
11	126	2006	2021	Carbapenem-resistant *A. baumannii*, blaOXA genes and OXA genes	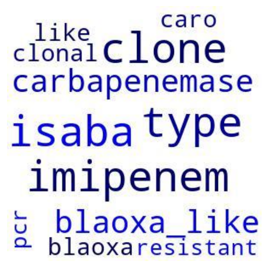	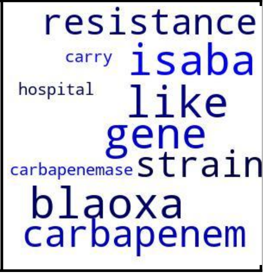

63	110	1998	2022	Nosocomial, clinical treatments and infections, healthcare	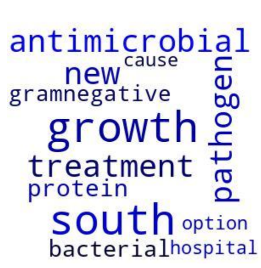	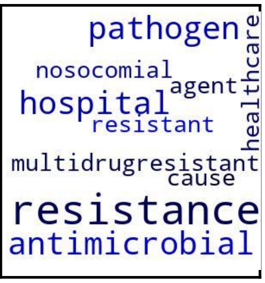

2.2 Assessing cluster labelling and model predictions

2.2.1 Automatic clustering labelling with LDA terms and terms from cluster centroids

To elucidate if the terms obtained with the LDA analysis and from the cluster centroids may be automatically used as labels of clusters, a person (curator) not involved in neither the clustering creation nor the clustering interpretation reviewed a 10% of randomly selected clusters (5, 13, 22, 24, 43, 49, 77, 78, 81, 87, 91, 109) and assigned a short phrase (label) to each cluster describing its thematic content. We obtained the percentage of LDA terms, terms from centroids, and the overlapped terms between both that appeared in the label. Supplementary Figure S3 depicts the percentage obtained by each type of term for each reviewed cluster.

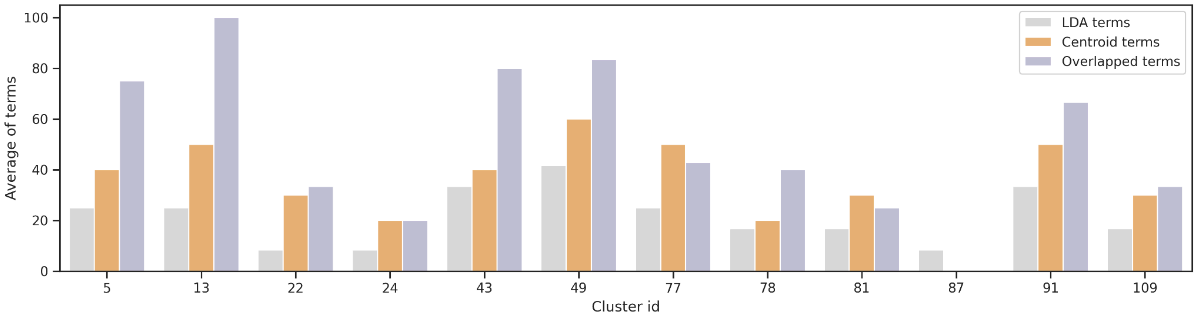

Supplementary Figure S3. Average of LDA terms, terms from centroids (Centroid terms), and overlapped terms between both (Overlapped terms) appearing in the manually assigned short phrase (label) of a 10% percent of randomly selected clusters: 5, 13, 22, 24, 43, 49, 77, 78, 81, 87, 91, 109.

The **Supplementary Table S4** (Table_S4.xlsx) containing the manually assigned labels, the terms and the percentage of them appearing in the manually assigned short phrase (label) is available at:

- https://github.com/laigen-unam/research-trends-ab or
- https://drive.google.com/drive/folders/18T6HwB9wdKVAn7VKs0nwUXKKvW9euD3K?usp=sharing

Column description

- **Cluster id**: cluster id, assigned by the K-means algorithm, of the 10% randomly selected clusters: 5, 13, 22, 24, 43, 49, 77, 78, 81, 87, 91, 109.
- **Total of publications**: total of publications grouped in the cluster by the K-means algorithm.
- **Total of titles reviewed to assign the label**: the number of titles reviewed by the curator to assign the label (short phrase) to the cluster describing the theme of the cluster.
- **Average of abstracts reviewed to assign the label**: the average of abstracts reviewed by the curator to assign the label (short phrase) to the cluster describing the theme of the cluster.
- **Manually assigned label**: a short phrase assigned by the curator describing the theme of the cluster.
- **Lemmas in label**: the lemmas obtained from the manually assigned label by applying a lemmatization task with the Stanza NLP library (Qi et al., 2020). Punctuation, symbols, and stop words were removed.
- **LDA terms**: the 12 terms automatically obtained by Mallet LDA analysis over the publications of the cluster.
- **LDA terms in label**: the LDA terms appearing in the lemmas of the label.
- **Percentage of LDA terms in label**: the percentage of LDA terms appearing in the lemmas of the label.
- **Terms from centroids**: the 10 terms automatically obtained from the cluster centroid of the cluster.
- **Terms from centroids in label**: the terms from cluster centroid appearing in the lemmas of the label.
- **Percentage of terms from centroids in label**: percentage of the terms from cluster centroid appearing in the lemmas of the label.
- **Overlapped terms between LDA terms and terms from centroids**: the overlapped terms between LDA terms and terms from cluster centroid.
- **Overlapped terms in label**: the overlapped terms appearing in the lemmas of the label.
- **Percentage of overlapped terms in label**: the percentage of the overlapped terms appearing in the lemmas of the label.

2.2.2 Cluster prediction using trained model

The clustering analysis of the 5511 publications allowed us to train a descriptive model that was used to predict the cluster for the 644 publications published from May 13th, 2022 to May 23rd, 2023. These publications were assigned to 100 different clusters, with a mean of 6.4 and median of 5 publications per cluster (Supplementary Figure S4). The distribution of publications by cluster shows that the majority of clusters attracted a few publications. In the Supplementary Table S5, we show the total of publications assigned to each cluster by the prediction of the model.

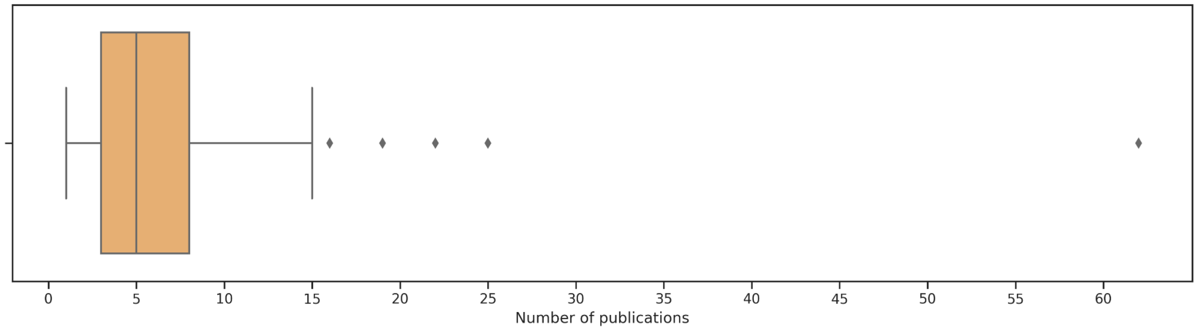

Supplementary Figure S4. Distribution of publications assigned to clusters by means of the prediction of the descriptive model.

Supplementary Table S5. The 644 publications, recovered from May 13th, 2022 to May 23rd, 2023, assigned to clusters by the prediction of the model. We show the manually assigned label of each cluster (Label) and the total of publications assigned to each cluster. The table is sorted in descending order by the total of publications assigned to each cluster.

**Cluster id**	**Total of publications assigned to each cluster**	**Label**
104	62	Multidrug-Resistant *A. baumannii* and Extensively Drug-Resistant *A. baumannii*
108	25	Carbapenem-resistant *A. baumannii* (CRAB)
16	22	Phage and bacteriophage
18	19	Antimicrobial activity of compounds
93	16	Biofilm formation and anti-biofilm
49	15	Vaccine and vaccine candidates
95	15	Virulence and virulence factors in *A. baumannii*
100	14	Inhibitor protein and inhibitor binding
54	13	Genomic analysis and genome sequences
87	13	Immunization and vaccine
39	12	Colistin resistance and heteroresistance
78	12	Cefiderocol against *A. baumannii*
48	11	Bacteraemia/bacteremia, risk factors, mortality, critically ill patients
52	11	Carbapenem-resistant *A. baumannii*, OXA carbapenemases, blaOXA
75	10	Mutants in different growth conditions
84	10	In vitro and in vivo studies/activity/efficacy
3	9	Biofilm and biofilm formation
19	9	Structure of capsular polysaccharides
21	9	Intensive care units (ICUs)
44	9	Ventilator-associated pneumonia (VAP)
106	9	Crystal structures and crystallization
110	9	Polymyxin and polymyxin in combination with other antimicrobials
6	8	General aspects of drug resistance of *A. baumannii*
46	8	Peptides
79	8	Concentrations and MICs
92	8	Efflux pumps, mainly AdeABC
2	7	Bacteriophages and phages, mainly lytic
9	7	Innate immune response
30	7	*A. baumannii* in cells, mainly epithelial cells
47	7	Antibiotic resistant and antibiotic resistant mechanism

70	7	In vitro antimicrobial combination and synergy/synergistic
76	7	Pneumonia caused by *A. baumannii*
96	7	Sulbactam in combination with other antimicrobials
98	7	Risk factors for colonization and in hospitals
107	7	Corrigendum, erratum, correction
22	6	Studies of plasmids
32	6	Hospital and nosocomial outbreaks
42	6	Pseudomonas aeruginosa and *A. baumannii*
60	6	Detection of *A. baumannii* (PCR, rapid detection)
61	6	Outer membrane proteins, mainly OmpA
63	6	Nosocomial, clinical treatments and infections, healthcare
65	6	Molecular epidemiology, sequence typing and sequence types
88	6	Epidemiology and molecular epidemiology in hospitals
101	6	Meropenem in combination with other antimicrobials
111	6	Tigecycline, tigecycline treatment and in vitro activity of tigecycline
12	5	Proteins, mainly membrane proteins, in proteomic analysis
24	5	Silver, silver nanoparticles, and silver nanocomposite
27	5	Ventriculitis and intraventricular/intravenous colistin
36	5	Pseudomonas aeruginosa and *A. baumannii*
51	5	*blaOXA* genes and *blaNDM* genes
53	5	Case reports of illness caused by *A. baumannii*
55	5	Different systems in *A. baumannii*, some with metals (copper, zinc)
56	5	Light, blue light and photodynamic
68	5	Survival and degradation of *A. baumannii* in different conditions
73	5	Burns units and wound infections
105	5	Minocycline in combination with colistin and polymyxin
26	4	Genetic studies, gene mutations (*gyrA*, *parC*)
57	4	Beta-lactam and beta-lactamase inhibitor
59	4	Animals, animal model and veterinary
66	4	OXA beta-lactamase and OXA carbapenemase in carbapenem resistance
69	4	Colistin-resistant mutations
71	4	Risk factors for mortality in bacteremia
97	4	Mouse model, mainly pneumonia and lung infection
102	4	Quorum sensing, biofilm and quorum quenching
1	3	Susceptibility testing, E-test, tigecycline susceptibility
4	3	Gene expression, several mentions of acid
10	3	*A. baumannii* in human body louse, meat, animals, extrahuman parts
20	3	Human serum and human serum albumin (HSA)
29	3	Environmental contamination and cleaning during outbreaks, mainly in hospitals and intensive care units
31	3	Complete genome studies
41	3	Outer membrane vesicles
45	3	Mechanisms of carbapenem resistance, mainly in hospitals
50	3	Iron and siderophores

74	3	Secretion system, mainly *vgrG*/VgrG
80	3	Analysis of DNA, mainly amplification and amplified analysis
90	3	Systematic review and meta-analysis
14	2	Different aspects of multidrug-resistant *A. baumannii*
33	2	Clones and international/european clones in hospitals
35	2	Lipopolysaccharide, loss of lipopolysaccharide, mainly in colistin resistance
38	2	Efflux pumps Ade-type (AdeABC, AdeR, AdeRS)
77	2	Aminoglycoside and rRNA methylase
82	2	Combination of antibiotics against *A. baumannii* especially colistin, rifampicin and imipenem
89	2	Surface-associated motility in *A. baumannii*
99	2	Typing methods: pulsed-field gel electrophoresis (PFGE), multilocus sequence typing (MLST)
11	1	Carbapenem-resistant *A. baumannii*, blaOXA genes and OXA genes
15	1	Polymyxin and metabolic/metabolomic studies
17	1	Carbapenem-resistant OXAs from hospitals
23	1	Resistant islands, mainly AbaR-type and AbGRI-type
25	1	Community-acquired infections, mainly pneumonia
28	1	Carbapenem resistance, OXA-type carbapenemase, blaOXA
40	1	Metallo-beta-lactamase (MBL), mainly in carbapenem-resistant *A. baumannii*
43	1	Species of *A. baumannii*, mainly Acinetobacter calcoaceticus-Acinetobacter baumannii complex
62	1	Class 1 and 2 integrons and gene cassettes
67	1	Pan-drug-resistant *A. baumannii* in Taiwan
72	1	Draft genome sequences and genome sequences
81	1	Colonies, opacity, translucent, phenotypes
86	1	Beta-lactamase, mainly ADC and AmpC
94	1	Sepsis caused by *A. baumannii*
109	1	Extended-spectrum beta-lactamase (ESBL) (VEB, PER)
112	1	Methicillin-resistant *Staphylococcus aureus* and *A. baumannii*

3 Supplementary References

- Arthur, D. & Vassilvitskii, S. (2007). K-means++: The Advantages of Careful Seeding. In Proceedings of the Eighteenth Annual ACM-SIAM Symposium on Discrete Algorithms, pp. 1027–1035, Society for Industrial and Applied Mathematics, Philadelphia.
- Qi, P., Zhang, Y., Zhang, Y., Bolton, J., & Manning, C. D. (2020). Stanza: A Python Natural Language Processing Toolkit for Many Human Languages. In Proceedings of the 58th Annual Meeting of the Association for Computational Linguistics: System Demonstrations, pp. 101–108, Online. Association for Computational Linguistics.
